# Crucial Role of IL1beta and C3a in the *In Vitro*-Response of Multipotent Mesenchymal Stromal Cells to Inflammatory Mediators of Polytrauma

**DOI:** 10.1371/journal.pone.0116772

**Published:** 2015-01-06

**Authors:** Nina-Emily Hengartner, Jörg Fiedler, Hubert Schrezenmeier, Markus Huber-Lang, Rolf E. Brenner

**Affiliations:** 1 Orthopedic Department, Division for Biochemistry of Joint and Connective Tissue Diseases, University of Ulm, Ulm, Germany; 2 Institute of Clinical Transfusion Medicine and Immunogenetics Ulm, German Red Cross Blood Transfusion Service Baden-Württemberg—Hessen and University Hospital Ulm, University of Ulm, Ulm, Germany; 3 Department of Orthopedic Trauma, Hand-, Plastic and Reconstructive Surgery, University of Ulm, Ulm, Germany; Wake Forest Institute for Regenerative Medicine, UNITED STATES

## Abstract

Multipotent mesenchymal stromal cells (MSC) exert immune-modulatory effects and support tissue regeneration in various local trauma models. In case of a polytrauma, high amounts of danger-associated molecular patterns are released, leading to a systemic increase of inflammatory mediators. The influence of such a complex inflammatory microenvironment on human MSC is mainly unknown so far. Therefore, we investigated the effects of a defined serum-free polytrauma “cocktail” containing ILͳbeta, IL6, IL8 and the anaphylatoxins C3a and C5a, in concentrations corresponding to those measured in the blood of polytrauma patients, on human MSC in vitro. The polytrauma cocktail induced directed migration of MSC with C3a representing its major soluble chemoattractive agent. Furthermore, the polytrauma cocktail and IL1beta upregulated the expression of MMP1 indicating a potential role of IL1beta to enhance MSC migration in the tissue context. COX2, PTGES and TSG6 were also found to be upregulated upon stimulation with the polytrauma cocktail or IL1beta, but not through other single factors of the polytrauma cocktail in pathophysiologically relevant concentrations. An RNA expression array of 84 inflammation-related genes revealed that both the polytrauma cocktail and IL1beta induced C3, CSF1, TLR3 and various chemokines without major qualitative or quantitative differences. These results indicate that IL1beta is a crucial mediator of the polytrauma cocktail in terms of immune-modulation and MMP1 expression. Thus, upon encountering the primary sterile, inflammatory milieu of a polytrauma, endogenous or systemically transfused MSC might be able to migrate to sites of injury, secrete TSG6 and PGE2 and to influence macrophage biology as observed in local trauma models.

## Introduction

A polytrauma (PT) is the most common cause of death in individuals below the age of 40 [1]. Early mortality within the first 24 h after trauma due to multiple severe injuries significantly differs from late mortality associated with systemic inflammatory response syndrome (SIRS) [2]. When tissues are severely damaged, massive amounts of danger-associated molecular patterns (DAMPs) such as heat-shock proteins, purine metabolites, membrane and matrix fragments as well as polynucleotides (DNA-/RNA-derived) are released [3]. As a consequence, various inflammatory mediators such as pro- and anti-inflammatory cytokines, complement and coagulation factors and proteins of the acute phase reaction are released in high amounts or subsequently activated potentially leading to SIRS as reviewed by Keel and Trentz [2]. Key inflammatory mediators often found to be increased in the plasma of PT patients early after injury are amongst others interleukin (IL) 1, IL6 and IL8 as well as the complement factors C3a and C5a [4–13].

In recent years, it became apparent that the numbers of multipotent mesenchymal stromal cells (MSC) in the blood significantly increases after injury such as hip or lower extremities fractures, acute myocardial infarction, muscle damage and severe tissue hypoxia [14–17]. Since the immunophenotype and differentiation potential of these MSC were shown to be similar to those of MSC isolated from the bone marrow [15], one explanation could be that MSC are systemically recruited from the bone marrow to various tissues. As MSC have been isolated from several other sources than bone marrow, their recruitment from the respective niches to sites of injury is conceivable as well [18–22]. If and how MSC affect the trauma response remains to be elucidated. Several studies, however, have shown that *ex vivo* expanded MSC injected into the circulation may engraft into both healthy and damaged tissues after they are initially trapped in the lung capillaries [23–26]. *In vitro* and *in vivo*, MSC have been shown to exert various immune-modulatory effects as reviewed by Yagi et al. and many others [27]. In the PT setting, it is not known so far if MSC are mobilized from the bone marrow or other tissues and whether they are sufficient in numbers to exert immune-modulatory effects. Theoretically, a future therapeutic strategy could be to administer MSC after a PT to reduce inflammation and to augment regeneration.

Systemic mobilization of MSC from their bone marrow niche as well as engraftment after intravenous administration requires cell migration depending on coordinated adhesion/detachment processes and differential matrix metalloproteinase (MMP) activity. Previous studies have shown that migration, adhesion to matrix proteins as well as MMP production are affected by high doses of the inflammatory cytokine IL1beta [28]. The systemic concentration of IL1beta after a PT, however, is much lower than usually employed in previous *in vitro* studies. If and how these pathophysiologically relevant concentrations as well as a “cocktail” of possibly interacting key pro-inflammatory mediators found in PT patients at an early stage of SIRS affect endogenous or transplanted MSC is largely unknown so far.

Macrophages are one of the central players in the early release of pro-inflammatory cytokines after PT. Various recent studies focusing on the MSC mediated immune-modulation of macrophages and the expression of immune-modulating factors by MSC have used interferon (IFN) γ or lipopolysaccharide (LPS) [29–31], both of which may not necessarily be present in the early phase after PT. If and how a primary sterile, inflammatory milieu after a PT affects MSC in terms of immune-modulation has not been investigated so far. Therefore, we defined a serum-free PT “cocktail” (PTC), qualitatively and quantitatively corresponding to some major cytokines and anaphylatoxins measured in the plasma or serum of multiply injured patients early (within 24 h) after trauma. Polytrauma studies in humans reporting IL1beta with 100–300 pg/ml, IL 6 with 100–800 pg/ml, IL8 with 70–150 pg/ml, complement factors C3a with 500–1000 ng/ml and C5a with 10–20 ng/ml were used as a rationale to define the cocktail [4–10]. We investigated the *in vitro* effects of this PTC and the relative contribution of its single factors on the migration of MSC, their expression of MMP and tissue inhibitors of MMP (TIMP) as well as their expression of immune-modulatory factors. Since the PTC and IL1beta alone exhibited most prominent effects, a PCR array of 84 genes related to inflammation was used to compare their effect on human MSC in a broader context.

## Materials and Methods

### Primary MSC isolation and culture

Human MSC were isolated from the bone marrow taken during routine surgical procedures (triple osteotomy) with informed consent of the patients according to the requirements of the Ethics committee at the University of Ulm. After a density gradient centrifugation (600 x g) of the bone marrow with Biocoll (Biochrome, Berlin, Germany), mononuclear cells were harvested from the interphase, washed with PBS and resuspended in basal medium which consisted of Dulbecco’s Modified Eagle Medium (DMEM) supplemented with 10% FCS, 100 U/100 µg/ml penicillin/streptomycin and 2 mM L-glutamine (all Biochrome). Mononuclear cells were cultured in 75 cm^2^ cell culture flasks at 37°C, 95% humidity and 5% CO_2_, after non-adherent cells had been washed off 24 h past isolation. To enhance MSC growth, 10 ng/ml fibroblast growth factor (FGF) 2 (Peprotech, Hamburg, Germany) were added to the medium. The medium was changed twice a week and MSC were split at a confluency of approximately 60%. Two days prior to experiments, FGF2 was taken off and MSC were cultured in basal medium. MSC of passages 3–5 were used for the experiments. The surface marker profile and differentiation potential of these MSC were examined for various cell preparations. MSC differentiated along osteogenic (positive staining for ALP and Alizarin Red S), adipogenic (positive staining for Oil Red O) and chondrogenic lineages (positive staining for COMP, Collagen type II, and proteoglycans by Alcian Blue) as exemplary shown for one donor in S1 Supporting Information. In the FACS analysis, more than 99% of MSC were CD45 negative and expressed the positive MSC markers CD90, CD73, CD29, CD166 and CD105 (all > 98%, S2 Supporting Information). A more extensive surface marker profile of MSC regularly isolated in the laboratory has recently been published [32].

### Analysis of MSC multipotency

For osteogenic and adipogenic differentiation, MSC were seeded at 5.2*10^3^/cm^2^ and 7.3*10^3^/cm^2^, respectively, and cultured in basal medium for 3 days. Medium was changed to osteogenic and adipogenic differentiation media, controls were cultured in basal medium. The adipogenic medium consisted of basal medium supplemented with 1µM dexamethasone, 0.45 mM isobutylmethylxanthine, 0.1 mM indomethacin (all Sigma-Aldrich), 5 µg/ml insulin, 5 µg/ml transferrin and 5 ng/ml sodium selenite. The osteogenic medium consisted of basal medium with 0.1 µM dexamethasone, 0.2 mM ascorbic acid (Fluka, Taufkirchen, Germany) and 10mM β-glycerophosphate (Sigma-Aldrich). The Oil Red O and Alizarin Red S stainings were performed according to standard protocols. Alkaline phosphatase activity was measured with the Leukocyte Alkaline Phosphatase Kit (Sigma-Aldrich) as directed by the manufacturer. For the chondrogenic differentiation, MSC were pelleted in micromass culture at 2 × 10^5^ MSC per pellet by centrifugation and cultivated in chondrogenic medium for 28 days. Basal medium was used as control. Chondrogenic differentiation medium consisted of DMEM with 4.5 g/L glucose and supplemented with 100 U/ml penicillin/streptomycin, 2mM L-glutamine, 1mM Na-pyruvate, 40 µg/ml L-proline (Sigma-Aldrich), 0.1µM dexamethasone, 0.2 mM ascorbic acid, 5 µg/ml insulin, 5 µg/ml transferrin and 5 ng/ml sodium selenite and 10 ng/ml TGFbeta3 (Peprotech). Alcian Blue staining was done according to standard protocols. Immunohistochemistry was performed on the paraffin-embedded micromass cultures with the collagen II antibody (neoLab, Heidelberg, Germany), the cartilage oligomeric matrix protein (COMP) antibody (kindly provided by Dr. Zaucke, Köln, Germany) and the DAKO LSAB2 Kit (Dako, Hamburg, Germany) as described before [33].

### FACS analysis

FACS analysis was performed as described earlier [32]. Briefly, 10^5^ MSC in 100 µl PBS were incubated with fluorochrome-labelled antibodies against CD90, CD29 (eBiosciences, San Diego, CA, USA), CD105 (BioLegend, San Diego, CA, USA), CD73, CD166, CD45 (BD Pharmingen, Heidelberg, Germany), C3aR (AbD Serotec, Puchheim, Germany) or the according isotype controls for 30 minutes in the dark on ice. After washing MSC twice with PBS, MSC were resuspended in 300 µl PBS and analyzed immediately on the FACSCalibur flow cytometer (Becton Dickinson, Heidelberg, Germany) with dual-laser technology and the CellQuest software V. 5 (Becton Dickinson). A minimum of 1.5*10^4^ cells were acquired.

### Exposure of MSC to cytokines and PTC

For cytokine stimulation, MSC were seeded at a density of 5.2*10^3^ cells/cm^2^ for all real-time PCR quantifications, TSG6 and PGE_2_ measurements in supernatant fluids. Since constitutive MMP9 and MMP1 expression as well as PGE_2_ levels in supernatants were found to be fairly low, MSC were seeded at a density of 5.2*10^4^ cells/cm^2^ for corresponding protein analysis. MSC were allowed to adhere in basal medium for 24h. Medium was then changed to serum-free conditions (DMEM supplemented with 5 µg/ml insulin, 5 µg/ml transferrin, 5 ng/ml sodium selenite (Roche, Mannheim, Germany), 1mM sodium pyruvate, non-essential amino acids, 100 U/100 µg/ml penicillin/streptomycin and 2 mM L-glutamine (all Biochrome)) and factors were added. The PTC consisted of 200 pg/ml IL1beta, 500 pg/ml IL6, 150 pg/ml IL8 (both BIOMOL, Hamburg, Germany), 500 ng/ml C3a and 10 ng/ml C5a (both Calbiochem, Darmstadt, Germany). All factors were also individually tested at the respective concentration of the PTC. In order to investigate concentration-dependent effects of IL1beta, IL1beta was also tested at 10 ng/ml for some of the analyses. In experiments, in which the binding of IL1beta to the IL1 receptor I (IL1RI) was inhibited to suppress IL1beta signaling, 2 µg/ml of the polyclonal goat anti-human IL1RI antibody (AF269, R&D Systems, Wiesbaden, German) was added to the medium prior to addition of IL1beta or the PTC. Polyclonal goat IgG (R&D Systems) was added at 2 µg/ml to control wells.

### Immunocytochemistry

In order to investigate IL1RI expression 10^4^ MSC were seeded per well on 4-well glass slides with polystyrene vessels (BD Falcon, Heidelberg, Germany) in basal medium. After over night adherence, serum-free medium was added 30min before staining. The culture slides were fixed in a 4% formaldehyde solution and MSC were stained with 2 µg/ml of the anti-human IL1RI antibody (AF269, R&D Systems) and the Dako LSAB+ System-HRP (K0690 Dako, Hamburg, Germany) according to the manufacturer’s protocol. Negative controls were stained with the IgG isotype control.

### RNA isolation and gene expression analysis

RNA was isolated with the RNeasy MiniKit (Qiagen, Hilden, Germany) from stimulated and unstimulated MSC 24 h after cytokine stimulation according to the manufacturer’s instructions. Reverse transcription was carried out with the OmniScript RT Kit (Qiagen). For quantitative real-time PCR, primers were used alongside the Power SYBR Green Master Mix and probes were used concomitantly with the Taqman Gene Expression Assay Master Mix (all Life Technologies, Darmstadt, Germany). The following primers and probes were employed in this study: MMP1 NM_002421.3, forward TTC GGG GAG AAG TGA TGT TC, reverse ATC TCT GTC GGC AAA TTC GT; cyclooxygenase 2 (COX2), NM_000963, forward CCC TTG GGT GTC AAA GGT AA, reverse GGC AAA GAA TGC AAA CAT CA; prostaglandin E synthase (PTGES) Hs01115610_m1; TNF-stimulated gene 6 protein (TSG6) Hs01113602_m1; MMP2 Hs01548727_m1; MMP9 Hs00234579_m1; tissue inhibitor of metalloproteinase (TIMP) 1 Hs00171558_m1; TIMP2 Hs00234278_m1; IL1 receptor antagonist (IL1RN); Hs00893626_m1; hypoxanthine phosphoribosyltransferase 1 (HPRT1) Hs02800695_m1. Acquisition and analysis of quantitative real-time PCR data was carried out with the StepOne reader and corresponding software (Life Technologies). RNA expression of additional pro-inflammatory cytokines was investigated with the RT^2^ Profiler PCR Array Human Inflammatory Response and Autoimmunity (Qiagen) as well as the ABI7000 reader and the 7000 system software (Life Technologies). If mRNA expression was below the detection limit, the Ct value was set to 40 in quantitative real-time experiments using primers or probes and to 35 in the RT^2^ Profiler PCR Array Human Inflammatory Response and Autoimmunity.

### MMP1, PGE_2_ and IL1RN detection

Pro-MMP1 was detected with the SensoLyte Plus 520 MMP-1 Assay Kit (AnaSpec, Freemont, CA, USA). PGE_2_ and IL1RN levels were measured with the Biotrend PGE_2_ Enzyme Immunoassay Kit (Biotrend Chemikalien, Köln, Germany) and the Human IL-1ra/IL-1F3 Quantikine ELISA Kit (R&D Systems, Wiesbaden, Germany) according to the manufacturer’s instructions. Fluorescence and absorbance values were measured on a Tecan Infinite M200 reader (Tecan, Crailsheim, Germany).

### TSG6 ELISA

TSG6 protein concentrations in cell culture media were determined by ELISA as previously described [24]. Briefly, the wells were coated with 100 µl/well and 10 µg/ml anti-TSG6 antibody (sc-65886, Santa Cruz, Heidelberg, Germany) in PBS over night at 4°C. Wells were washed and blocked with blocking buffer (PBS + 0.05% Tween + 0.5% BSA) for 1 h at room temperature. Afterwards, wells were washed again. Recombinant human TSG6 (2104-TS-050, R&D Systems) was used as standard, ranging from 4800 pg/ml to 75 pg/ml. Standards, negative controls or samples (each at 100 µl/well) were added and incubated for 2 h at room temperature. After another washing step, 100 µl/well biotinylated anti-TSG6 antibody (BAF2104, R&D Systems), diluted at 0.5 µg/ml in blocking buffer, was added and incubated for 2h at room temperature. Wells were washed and 100 µl/well streptavidin-HRP (R&D Systems), diluted in blocking buffer as directed by the manufacturer, were added and incubated for 20 min at room temperature in the dark. Wells were washed again and 100 µl/well of substrate solution (DY999, R&D Systems) were added. After 10–30 min incubation at room temperature in the dark, 50 µl/well stop solution (DY994, R&D Systems) were added and absorbance was immediately measured at 450 nm on the Tecan Infinite M200 reader.

### Zymography

Gelatin zymography was used for the detection of MMP2 and MMP9 on a protein level and the corresponding activity. Supernatant fluids were mixed 1:2 with non-reducing Zymogram sample buffer (BioRad, Munich, Germany). Samples were loaded onto 0.75 mm thick, 7.5% polyacrylamide gels (all components Carl Roth, Karlsruhe, Germany) containing 2 mg/ml gelatin (Merck, Darmstadt, Deutschland). Electrophoresis was carried out on the Mini-PROTEAN Tetra Cell System (Bio-Rad). Afterwards, gels were washed twice for 15 min in Zymogram renaturation buffer and incubated over night in Zymogram development buffer at 37°C (both Bio-Rad). Staining with Coomassie solution and subsequent destaining revealed clear bands originating from MMP activity. Pictures of gels were taken and analyzed with the Gel Doc XR+ System and the Quantity 1D Analysis Software (both Bio-Rad).

### Chemotaxis of MSC

Chemotaxis assays were performed in a modified Boyden chamber employing polycarbonate filters with 8 µm pores (both Neuro Probe, Gaithersburg, MD, USA) between the upper well holding the MSC and the lower well holding the chemotactic factors as described previously [34–36]. Chemotactic activity of MSC was analyzed during exposure to the PTC or its single components at the following concentrations: 200 pg/ml IL1beta, 500 pg/ml IL6, 150 pg/ml IL8, 500 ng/ml C3a and 10 ng/ml C5a. Platelet-derived growth factor (PDGF) BB at a concentration of 10 ng/ml was used as a positive control (not shown). For a negative control, DMEM was used. After MSC had been trypsinized, washed and resuspended in serum-free DMEM, 10^4^ MSC were filled into the upper wells of the Boyden chamber. In case of inhibition of the C3a—C3aR interaction, the C3aR antagonist SB 290157 (Merck, Darmstadt, Germany), dissolved in DMSO, was added to the MSC suspension at a concentration of 10 µM 30 min prior to chemotaxis assays. DMSO was added to controls at the same dilution. Before addition to the Boyden chamber, MSC were washed once in DMEM. After a 4 h incubation period, filters were taken out and adherent, non-migrated cells on the upper side of the filter were scraped off using a rubber scraper. Migrated MSC on the lower side of the filter were stained with Giemsa solution (Merck, Darmstadt, Germany) after fixating them in a 4% formaldehyde solution. Migrated cells were counted at 20x magnification (Reichert Jung Polyvar microscope). For checkerboard analysis, the migration activity of MSC under basal conditions, with the chemoattractant only in the lower and in both the upper and lower compartment was determined. Chemotaxis was always analyzed in quadruplicates.

### Statistics

Data are presented as scatter dot plots with the mean from up to 7 independent experiments with MSC from different donors. Statistical significance was determined with one-way ANOVA and a Dunnett’s post-test or Student’s t-test using GraphPad Prism 5 (GraphPad Software), when donor number was ≥3. Data from the RT^2^ Profiler PCR Array Human Inflammatory Response and Autoimmunity are presented as mean from 3 independent donors. Analysis was performed with the RT Profiler PCR Array Data Analysis version 3.5 online (http://pcrdataanalysis.sabiosciences.com/pcr/arrayanalysis.php), which uses a Student’s t-test for determination of significance. Differences were considered significant when the p-value was <0.05 and marked with *.

## Results

### PTC and C3a alone induce MSC migration

**Figure 1:**
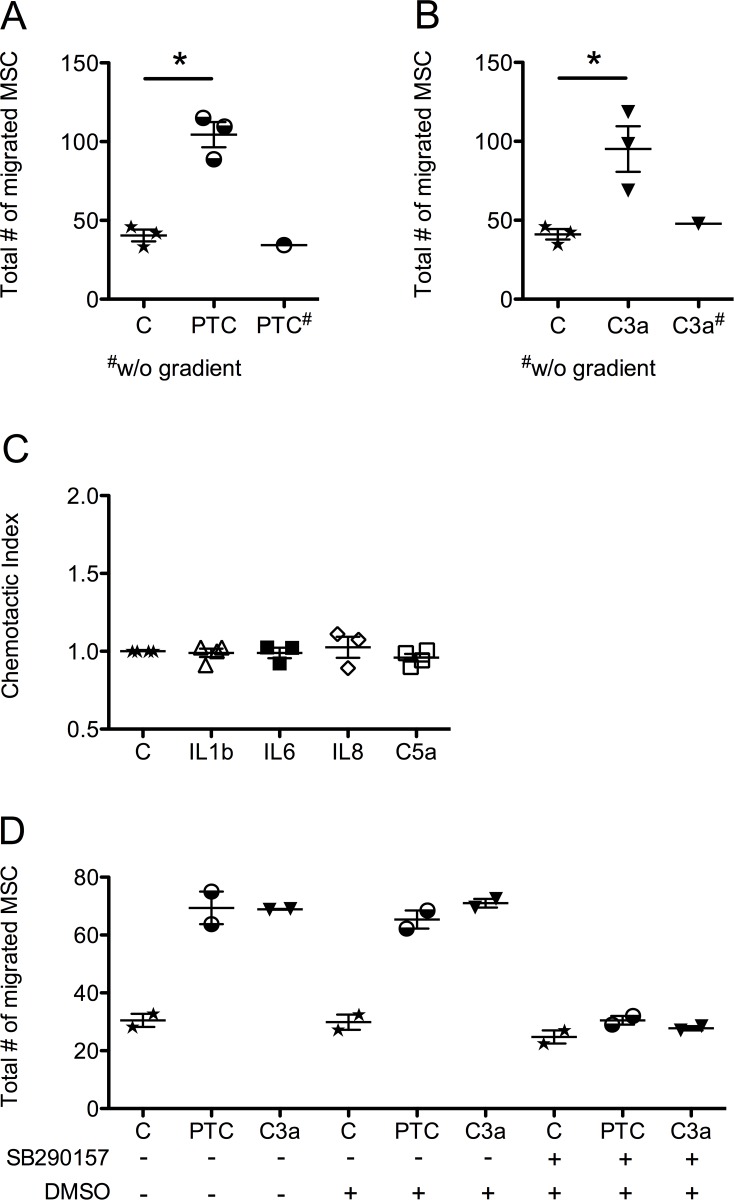
Chemotactic response of MSC towards the PTC and its individual components. In-vitro migration was analyzed in a modified Boyden chamber. Migration of MSC towards A) PTC B) C3a C) IL1beta, IL6, IL8 and C5a without prior treatment and D) PTC or C3a upon treatment with 10 µM of C3a inhibitor SB 290157 compared to controls is shown. Scatter plots and mean number of total migrated cells (A, B and D) or the mean chemotactic index (C) from up to 3 donors are presented. Significance was calculated using Student’s t-test; p*<0.05. For one donor, PTC (A) and C3a (B) were filled in both upper and lower wells (without gradient) to test if migration was due to chemotaxis or generally enhanced migratory behavior (chemokinesis).

In the chemotaxis experiments, we found that the PTC significantly increased migration in MSC when compared to control. A checkerboard analysis revealed that the PTC clearly induced directed cell migration (Fig. 1A). After individually testing each component of the PTC in the chemotaxis assays, we observed that C3a significantly stimulated MSC migration in pathophysiologically relevant concentration as shown in Fig. 1B. This effect was only present in the case of a concentration gradient, confirming a true chemotactic effect (Fig. 1B). IL1beta, IL6, IL8 and C5a, however, did not induce a directed migration of MSC, when applied in the respective concentration of the PTC, as the chemotactic indices show in Fig. 1C. A FACS analysis showed that 87.9% of the CD105+/CD73+ MSC also expressed the C3aR, of which 99.8% were CD45 negative. The C3a or PTC mediated chemotaxis could be completely inhibited by the C3aR antagonist SB 290157 (Fig. 1D).

### PTC and IL1beta alone result in enhanced MMP1expression of MSC

**Figure 2:**
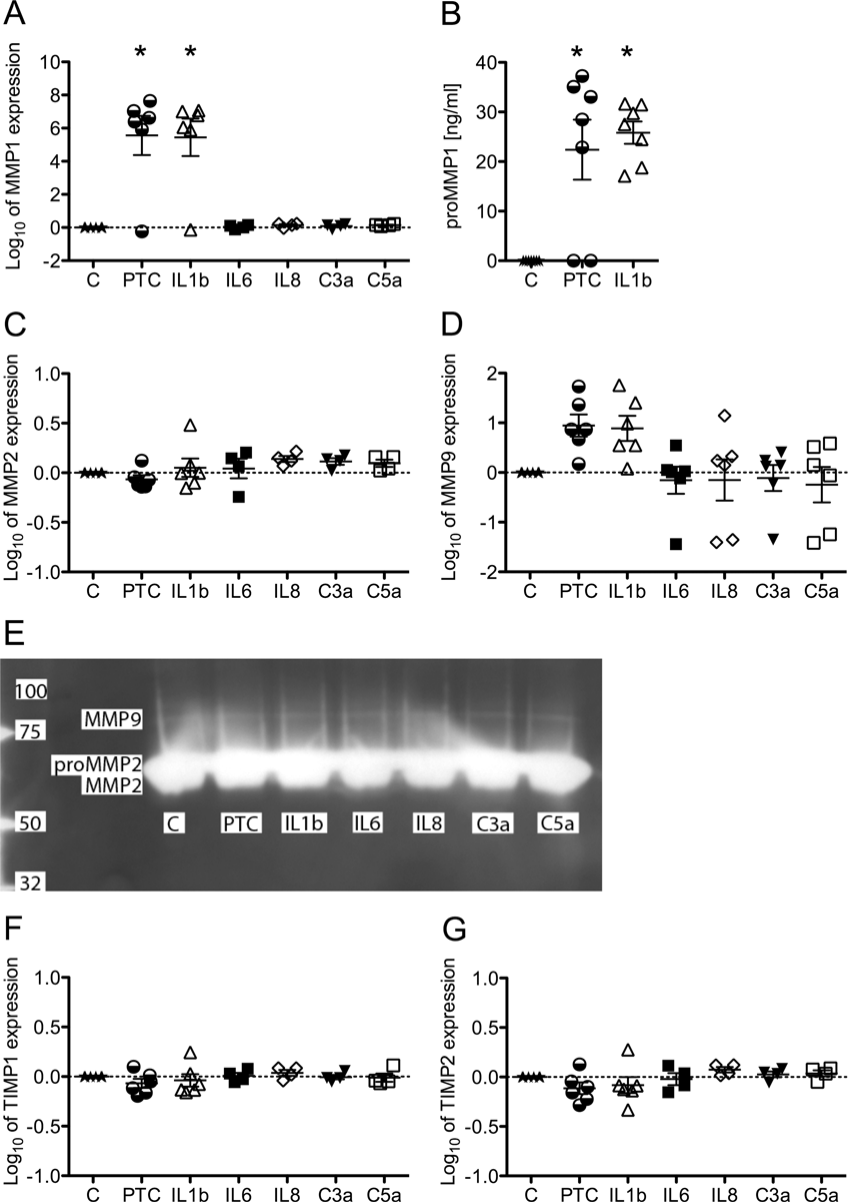
Influence of the PTC and its individual components on the expression of MMP and TIMP in MSC. Expression levels of A) MMP1, C) MMP2, D) MMP9, F) TIMP1 and G) TIMP2 were measured with quantitative real-time PCR and normalized to those of HPRT1 following total RNA isolation 24 h post stimulation from MSC seeded at 5.2*10^3^ cells/cm^2^ and stimulated with the PTC, its individual components or left untreated. B) Pro-MMP1 detection with the SensoLyte Plus 520 MMP-1 Assay Kit and E) Zymography from supernatants 24 h post stimulation from MSC seeded at 5.2*10^4^ cells/cm^2^ and stimulated with the PTC, its individual components or left untreated 24 h post stimulation. Scatter plots and mean from up to 7 donors are presented. Significance of regulation was determined with one-way ANOVA followed by a Dunnett’s post-test; *p<0.05.

Quantitative analyses of the mRNA expression revealed that MMP1 expression, which was observed to be below detection levels in unstimulated MSC, was significantly up-regulated in MSC when stimulated with the PTC or IL1beta (Fig. 2A). Stimulation with single IL6, IL8, C3a or C5a in the respective concentration of the PTC did not induce any up-regulation of MMP1 (Fig. 2A). On the protein level, pro-MMP1 expression was also up-regulated upon stimulation with the PTC or IL1beta (Fig. 2B). MMP2 mRNA was found to be constitutively expressed in MSC and not significantly regulated by the PTC or single IL1beta, IL6, IL8, C3a or C5a exposure as seen in Fig. 2C. Gelatin zymography revealed that the pro-enzyme MMP2 and MMP2 are found in supernatant fluids from MSC irrespective of PTC or cytokine stimulation as seen in Fig. 2E. MMP9 mRNA expression was observed to be either very low or below detectable levels in native MSC and tended to increase upon stimulation with the PTC or IL1beta as seen in Fig. 2D. Exposure to single IL6, IL8, C3a or C5a did not induce a higher MMP9 mRNA expression (Fig. 2D). In the gelatin zymography, MMP9 enzyme activity could barely be found in supernatants from untreated MSC and MSC treated with the PTC or its respective single components (Fig. 2E). The mRNA expression of the MMP inhibitors TIMP1 and TIMP2 remained mostly unchanged upon stimulation with IL6, IL8, C3a or C5a (Fig. 2F and 2G).

### PTC and IL1beta alone induce MSC expression of key immune-modulatory factors, such as COX2 and TSG6

**Figure 3:**
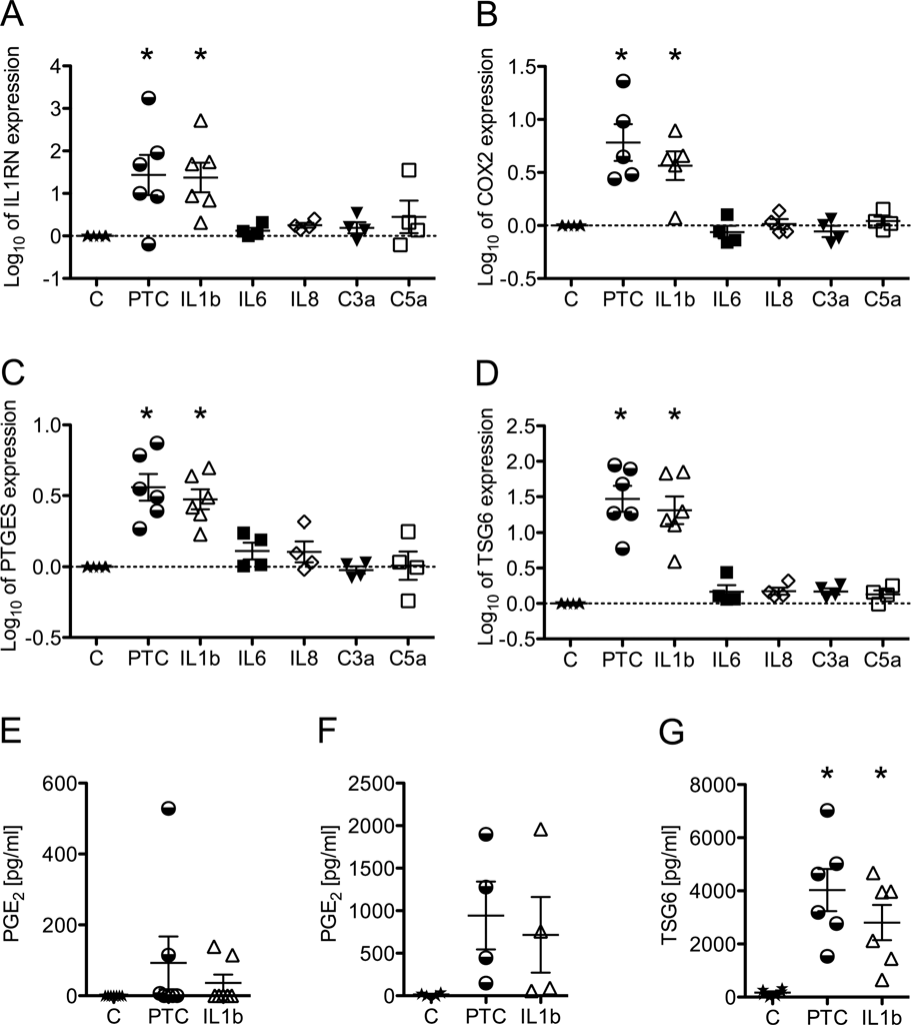
Influence of the PTC and its individual components on the expression of immune-modulatory factors in MSC. Expression levels of A) IL1RN, B) COX2, C) PTGES, and D) TSG6 were measured with quantitative real-time PCR and normalized to those of HPRT1 following total RNA isolation 24 h post stimulation from MSC seeded at 5.2*10^3^ cells/cm^2^ and stimulated with the PTC, its individual components or left untreated. E and F) PGE2 detection with the Biotrend PGE^2^ Enzyme Immunoassay Kit in supernatants 24 h post stimulation from MSC seeded at 5.2*10^3^ (E) or 5.2*10^4^ cells/cm^2^ (F) and stimulated with the PTC, its individual components or left untreated 24 h post stimulation. G) TSG6 ELISA with supernatants 24 h post stimulation from MSC seeded at 5.2*10^3^ cells/cm^2^ and stimulated with the PTC, its individual components or left untreated. Scatter plots and mean from up to 7 donors are presented. Significance of regulation was determined with one-way ANOVA followed by a Dunnett’s post-test; *p<0.05.

On the mRNA level of MSC, we found IL1RN, which binds to IL1R on the cell surface with subsequent inhibition of the IL1beta signaling pathway, to be upregulated from a low basal level of expression upon stimulation with the PTC (318-fold) or IL1beta (107-fold) and to remain unchanged after stimulation with IL6, IL8, C3a and C5a in concentrations used in the “cocktail” (Fig. 3A). IL1RN protein in supernatants of unstimulated and stimulated cultures, however, were below the detectable level of 30 pg/ml. COX2, the enzyme converting arachidonic acid to prostaglandin H_2_, was significantly upregulated about 9-fold by the PTC and about 4-fold by IL1beta, while IL6, IL8, C3a and C5a were ineffective in regard to the COX2 expression (Fig. 3B). PTGES, which converts PGH_2_ into the immune-modulatory factor PGE_2_, was significantly augmented in MSC stimulated with the PTC (4-fold) or IL1beta (3-fold), but did not rise upon stimulation with the single factors IL6, IL8, C3a and C5a (Fig. 3C). On a metabolite level of MSC, we observed that PGE_2_ was up-regulated upon PTC or IL1beta stimulation in only a few donors, when seeded at low cell density (Fig. 3E), and in most donors when confluently seeded (Fig. 3F).

TSG6, which induces macrophages to produce less inflammatory mediators, was found to be significantly up-regulated in MSC on RNA level upon stimulation with the PTC (43-fold) or IL1beta (32-fold), but remained unchanged when stimulated with IL6, IL8, C3a or C5a (Fig. 3D). Also, TSG6 protein was significantly upregulated in all donors when seeded in low cell density (Fig. 3G). The IL1beta-induced up-regulation of PTGES and TSG6 was found to be concentration-dependent, as the concentration of 10 ng/ml IL1beta (exceeding by far systemic levels in PT) induced a more pronounced response on the mRNA as well as the respective metabolite (PGE_2_)- and protein (TSG6)-level (S3A–C Supporting Information).

### PTC and IL1beta alone induce a similar MSC chemokine and cytokine expression pattern

**Figure 4:**
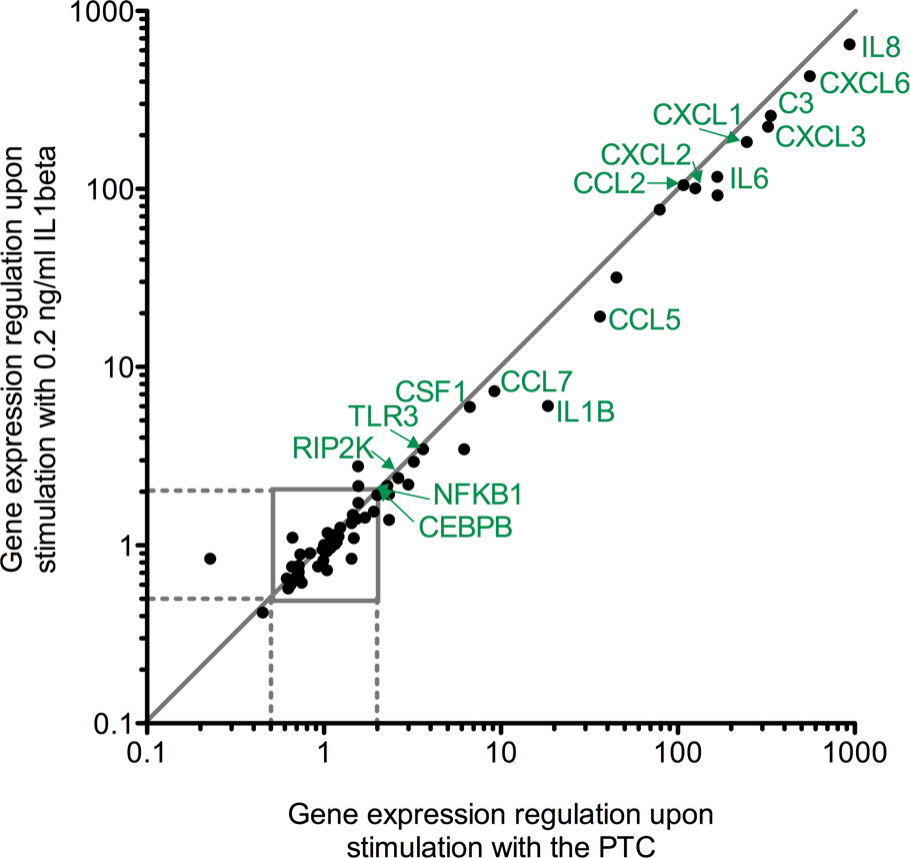
Influence of the PTC or IL1beta alone on the expression of 84 inflammatory factors in MSC. Total mRNA was isolated 24 h post stimulation from MSC seeded at 5.2*10^3^ cells/cm^2^ and stimulated with the PTC, IL1beta or left untreated. Quantitative real-time PCR with the RT^2^ Profiler PCR Array Human Inflammatory Response and Autoimmunity was performed. Analysis was performed with the RT Profiler PCR Array Data Analysis version 3.5 online (http://pcrdataanalysis.sabiosciences.com/pcr/arrayanalysis.php), which uses a Student’s t-test to determine significant gene expression fold changes in stimulated MSC compared to controls, p<0.05. Dots represent the mean from 3 independent donors. Genes that were significantly and more than two-fold up-regulated in both PTC and IL1beta treated MSC are marked with their gene name.

Upon stimulation of human MSC with the PTC, the chemokines and pro-inflammatory cytokines IL8, CXCL6, C3, CXCL3, CXCL1, CXCL5, IL6, CXCL2, CCL2, CCL5, IL1BETA, CCL7, CSF1, IL1A, TLR3, RIPK2, CEBPB and NF-κB were significantly and more than two-fold up-regulated as assessed by a gene expression array. MSC stimulated with IL1beta revealed a significant and more than two-fold up-regulation of IL8, CXCL6, C3, CXCL3, CXCL1, IL6, CXCL2, CCL2, CCL8, CCL5, IL1BETA, CCL7, CSF1, TLR3, RIPK2, CEBPB and NF-κB. In contrast, a more than twofold and significant down-regulation upon PTC or IL1beta exposure was not found. Genes that were significantly and more than two-fold upregulated in MSC by both PTC and IL1beta are marked with the respective name to the dot as shown in Fig. 4. Since the dots lie very close to the y = x line and are only minimally shifted to the PTC side, it is assumed that IL1beta alone and the PTC induce mostly the same genes at the same level, with the PTC having a marginally additive induction effect. When comparing the physiologically relevant concentration of IL1beta with the supraphysiologic concentration of 10 ng/ml, we found no major concentration-dependent effect as 10 ng/ml and 0.2 ng/ml IL1beta induced approximately the same genes in the array (S4 Supporting Information). The complete real-time PCR array data are found in the S5 Supporting Information.

### Inhibition of IL1beta effects by the addition of an anti-IL1RI antibody

**Figure 5:**
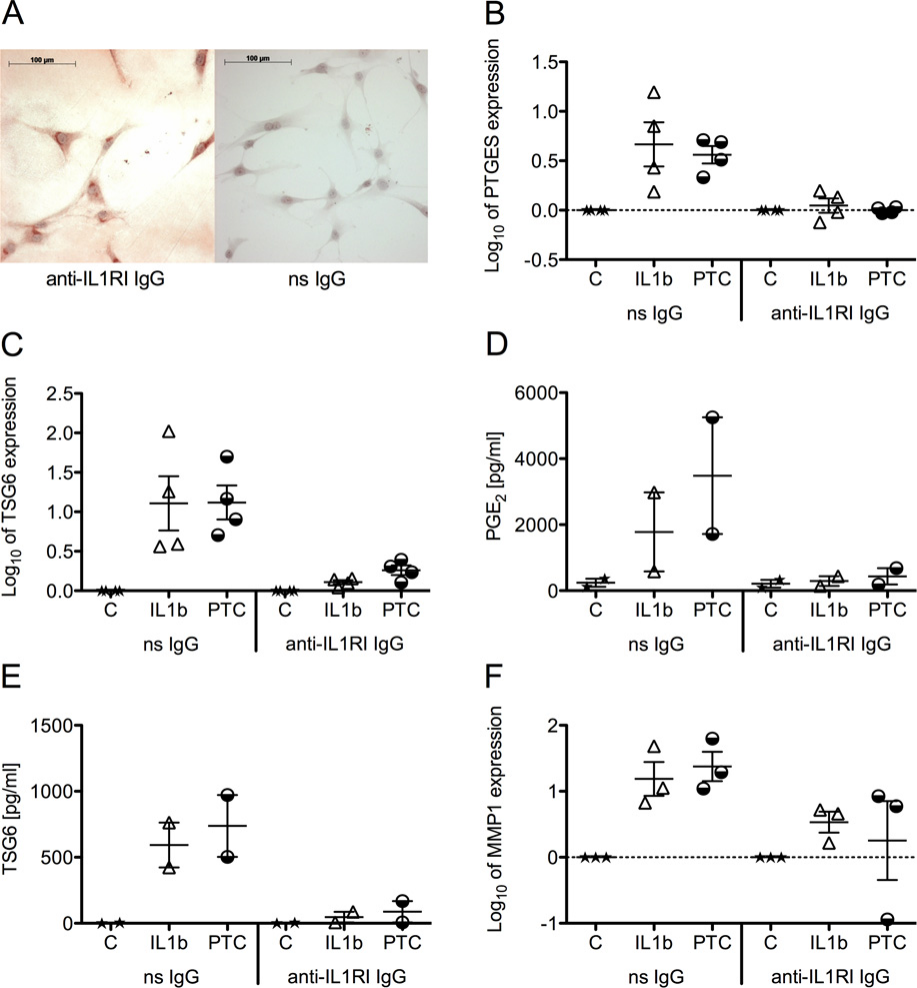
Expression of IL1RI in native MSC and inhibition of the IL1beta mediated regulation of gene expression in PTC or IL1beta stimulated MSC. A) MSC on glass slides were stained with an anti-IL1RI antibody (left) or the non-specific IgG isotype control (right). Gene expression levels of B) PTGES, C) TSG6 and F) MMP1 were measured with quantitative real-time PCR and normalized to those of HPRT1 following total RNA isolation 24 h post stimulation. D) PGE^2^ and E) TSG6 levels in supernatants were detected with the Biotrend PGE2 Enzyme Immunoassay Kit and TSG6 ELISA. MSC were seeded at 5.2*10^3^ cells/cm^2^ and stimulated with the PTC or IL1beta after the anti-IL1RI antibody or the non-specific (ns) IgG isotype control had been added. Scatter plots and mean from up to 4 donors are presented.

The precedent experiments suggested that IL1beta is a crucial mediator of the PTC. Staining of MSC with an anti-IL1RI antibody revealed that MSC constitutively express the IL1RI (Fig. 5A, left), while the isotype control showed no positive staining (Fig. 5A, right). The IL1beta mediated effects in the cell culture were then inhibited with the anti-IL1RI antibody and compared to the effects of the isotype control. We observed that, upon addition of the anti-IL1RI antibody to the serum-free medium before adding IL1beta or the PTC, the IL1beta and PTC mediated up-regulation of PTGES and TSG6 was completely inhibited (Fig. 5B and C). Likewise, the PGE_2_ levels in supernatants were 6- and 8-fold reduced in IL1beta and PTC stimulated cultures, respectively, when the anti-IL1RI antibody was added (Fig. 5D). Accordingly, TSG6 protein fell 12.6- and 9.6-fold in these cultures (Fig. 5E). The up-regulation of MMP1 mRNA was markedly reduced upon addition of the anti-IL1RI antibody as well (Fig. 5F).

## Discussion

Since PT patients carry a high risk of developing SIRS, great efforts are made to find means for early diagnosis and reduction of the systemic inflammation after PT. The potential of MSC to engraft in injured tissue especially in the lungs, and to exert various immune-modulatory effects, suggests that intravenous administration of MSC in PT patients might have promising therapeutic effects [24, 26, 31, 37, 38]. Therefore, we investigated in this *in vitro* study, if and how a PTC, simulating various cytokine concentrations measured in the serum of PT patients [4–10], affects MSC in terms of migration activity, MMP expression and expression of immune-modulatory factors. Furthermore, these effects were compared to the single components of the PTC.

MSC used in this study were able to differentiate into osteoblasts, adipocytes and chondrocytes. More than 99% of the MSC expressed the surface markers CD73, CD105, CD29 and CD166, while less than 1% expressed the hematopoietic marker CD45. Therefore, the MSC used in the experiments did not contain relevant amounts of hematopoietic cells. In the chemotaxis experiments, C3a and the PTC were found to have a significant chemotactic effect on human MSC. Since C3aR expression was found on almost 88% of the CD73, CD105 positive, CD45 negative MSC population, no specific subpopulation of MSC migrating towards C3a is assumed. As the other components of the PTC failed to induce chemotaxis of MSC *in vitro* and the chemotaxis towards the PTC was completely abolished by the C3aR antagonist SB290157 [39], we conclude C3a to be the major chemotactic agent of the PTC. The C3a-induced chemotactic effect on MSC is further supported by a study, in which C3a also induced a chemotactic effect at concentrations higher than 100 ng/ml [40]. Since C3a is highly abundant in severely traumatized patients [8–10], a potential role of C3a for the recruitment of MSC from their bone marrow niche into the circulation could be supposed. The perception of IL1beta not to be involved in migration of MSC is underpinned by a different study, in which comparable concentrations of IL1beta were used [41]. Only at extremely high concentrations (e.g. 25 ng/ml), that are by far not reached systemically after trauma, some stimulation of MSC migration through IL1beta has been reported [28]. Likewise, other studies have proposed some chemoattractive effects of IL6 and IL8 on MSC *in vitro*, at high concentrations of 10 and 100 ng/ml for IL6 [42, 43], at 50 ng/ml for IL8 [44]. While C5a did not have a chemotactic effect at systemically relevant concentrations, we observed a chemoattractive effect on MSC at higher concentrations, which is also reported by other studies at concentrations of 100 to 1,000 ng/ml [40, 45]. Nevertheless, locally released C5a at sites of injury might reach concentrations that may contribute to MSC recruitment.

Upon stimulation of MSC with the PTC and IL 1beta, gene expression of MMP 1 as well as the immune-modulatory factors IL1RN, COX2, PTGES and TSG6 were found to be up-regulated. When comparing the PTC with the single IL1beta stimulus, significant differences in the expression rate of MSC for MMPs and immune-modulatory factors were not observed. Since IL6, IL8, C3a and C5a as single factors failed to regulate the defined immune-modulatory factors or MMP1 and since upregulation of TSG6, PTGES and MMP1 gene expression as well as TSG6 protein and PGE_2_ through the PTC could be reduced or completely inhibited by blocking the IL1RI, we conclude that IL1beta is the crucial mediator of the PTC in terms of immune-modulation and MMP1 expression. The MSC-PCR array analysis of 84 inflammatory factors, in which all inflammatory factors up-regulated by the PTC were also up-regulated by IL1beta, further supports this notion. Since all MSC expressed the IL1R in the immunostaining, we do not assume a specific subpopulation being responsible for the IL1beta mediated effects detected.

As stated before, we suggest IL1beta to play a major role in MMP1 regulation of MSC. For the first time, we could show that IL1beta, at concentrations systemically present early after a PT, significantly up-regulates MMP1 mRNA and proMMP1 levels in supernatant fluids of MSC. The constitutive high expression of MMP2 and low expression of MMP9 found in this study is supported by Ries et al. [46]. At a higher concentration of IL1beta than employed in this study, they further observed an up-regulation of MMP 9 going in line with a facilitated migration through an endothelial barrier like matrix [46]. The IL1beta-induced MMP1 expression reported in this study at pathophysiologically relevant concentrations might promote the cleavage of interstitial collagen types I, II and III as well as other extracellular matrix components [47] and enable MSC to migrate into connective tissue at sites of tissue damage.

With regard to the human inflammatory response, we found 18 genes in a respective PCR array to be significantly and more than two-fold up-regulated upon stimulation of MSC with the PTC. Most of them code for factors that are primarily responsible for the recruitment of neutrophils, monocytes and macrophages. If MSC might be capable to recruit neutrophils and to exert immune-modulatory effects on them remains to be elucidated. Besides the IL6-mediated down-regulation of neutrophil apoptosis through MSC, not much is known how MSC affect neutrophil biology [48].

More evidence, however, is available on how MSC affect macrophages. Nemeth et al. found that LPS activated MSC secrete PGE_2_, which in turn induced macrophages to produce IL10 [31]. Based on a murine cecal puncture ligation model of sepsis, the authors suggested the observed reduction in mortality of septic mice after intravenous administration of MSC to be due to the IL10 release by PGE_2_ reprogrammed macrophages [31]. This PGE_2_ dependent, immune-modulatory effect of MSC on macrophages has been reported several times [49–51]. Choi et al. found that activated MSC release TSG6 which binds to CD44 on tissue resident macrophages, thereby reducing TLR2/NF-κB signaling and TNF secretion. The TSG6-induced decrease in TNF production resulted in diminished signs of zymosan-induced mouse peritonitis [52]. Furthermore, Qi et al. demonstrated that TSG6 released from MSC improved wound healing by limiting macrophage activation, inflammation and fibrosis [53].

Since we found the PTC and its crucial component IL1beta to up-regulate COX2, PTGES and the metabolite PGE_2_ as well as TSG6, we speculate that MSC administered in the primary sterile, inflammatory milieu after PT might affect macrophages via the secretion of PGE_2_ and/or TSG6. As IL1RN protein could not be detected in a concentration exceeding 30 pg/ml, though upregulated on gene expression level, when MSC were exposed to the PTC, we assume IL1RN not to be primarily involved in the immune-modulatory function of MSC under those PT conditions. It is also tempting to speculate, that TSG6 plays a more robust role in the PT setting than PGE_2_, as the PTC-induced up-regulation of PGE_2_ seemed to depend on cell-cell contact as it was only consistently up-regulated in MSC, which were seeded at a higher cell density. In contrast, TSG6 up-regulation seemed to be cell-cell contact independent in this study. Very recently, it has been reported that compaction of MSC into spheres as described after systemic application for cell therapy induces IL1-signaling which itself enhances the secretion of PGE_2_ and TSG6 as modulators of inflammation and immunity [54]. Our data indicate that, in the presence of clinically relevant concentrations of IL1beta either alone or in combination with other pro-inflammatory mediators of SIRS, a 3-dimensional aggregation of MSC is not necessary to induce TSG6 but maybe necessary for PGE_2_ production.

A first study has suggested the systemic transplantation of MSC in a rat model of femur fracture and hemorrhagic shock to have some positive therapeutic effects [55]. Further studies, however, are necessary to test these promising results in a more complex PT model as recently described [56]. Moreover, our results raise the question whether “*in vitro* priming” of MSC through IL1beta could speed-up and enhance immune-modulatory effects after transplantation *in vivo*. This might be of special relevance in trauma situations without any significant systemic inflammatory response.

As there is no time to grow autologous MSC after a PT, allogeneic MSC would have to be used in PT patients. A clinical trial investigating the effect of allogeneic MSC in patients with refractory systemic lupus erythematosus has found no serious adverse effects, but an improvement in disease activity, serological markers and renal function [57]. Another clinical study showed that independent of the donor the application of *in vitro* expanded MSC could be an effective therapy for patients with steroid-resistant, acute GVHD [58]. Furthermore, MSC from human source have been shown to persist for long times in a xenogenic environment after being transplanted indicating a lack of immune response by the host against MSC [59]. In the PT setting, blood banks could thaw cryo-presserved GMP-conform MSC upon a call from the emergency room, possibly prime them shortly with IL1beta and have them ready for administration within the first day after PT. In this scenario large-scale expanded cryopreserved cells would be used immediately after thawing without additional expansion. If the MSC are given within the acute (≤24 h) or subsequent primary phase of intensive care, they might have a chance to prevent full development of SIRS or to ameliorate its course. Nevertheless, many questions concerning a translation of such a strategy into clinical application remain open. Therefore, well-designed studies investigating the optimal culture conditions and the minimal necessary time for *in vitro* preconditioning as well as the overall therapeutic effects of systemic administration of MSC in animal models of PT should be the next steps to assess a potential benefit *in vivo* that might be clinically relevant.

## Conclusions

Upon *in vitro*-stimulation of MSC with a PTC consisting of IL1beta, IL6, IL8, C3a and C5a, we found IL1beta to be the crucial mediator in regulation of MMP 1 and immune-modulatory gene expression and C3a to be a mediator of chemotaxis. Thus, under PT conditions with abundant generation of C3a, this anaphylatoxin might be important for the recruitment of MSC from their physiological niches. The IL1beta mediated up-regulation of MMP 1 expression indicates that in the PT context IL1beta possibly facilitates interstitial migration of endogenously recruited MSC or systemically transplanted MSC within tissues. Furthermore, IL1beta-induced up-regulation of TSG6 and PGE_2_ in MSC *in vitro* suggests a potential role of *in vivo* administered MSC to influence macrophages to produce less TNF and more IL10. Further studies have to prove this concept under *in vivo* circumstances, which are by far more complex and comprise numerous additional factors such as TNFalpha, HMGB1 or other DAMPs not included in the simplified *in vitro*-model used in this study. Nevertheless, systemic administration of MSC after PT may represent a promising therapeutic option for the future.

## Supporting Information

S1 Supporting Information Differentiation potential of MSC. MSC were cultured with A) osteogenic differentiation medium (ODM), B) adipogenic differentation medium (ADM) or C) chondrogenic differentiation medium (CDM). A) Positive osteogenic differentiation as evidenced by positive staining for ALP (red dye deposit) and Alizarin S (red: calcium deposition). B) Positive Oil Red O staining indicates adipogenic differentiation (red: stained lipid droplets). C) Positive chondrogenic differentiation as demonstrated by positive immunohistochemical staining for cartilage oligomeric matrix protein (COMP) and collagen type II and positive Alcian Blue staining.
(JPG)

S2 Supporting Information Flow cytometric analysis of cultured MSC for the expression of CD45, CD90, CD73, CD105, CD29, CD166. Exemplary percentages of marker carrying MSC from 2 donors are depicted.
(PDF)

S3 Supporting Information Comparison between 0.2 and 10 ng/ml IL1beta stimulation on MSC. A) mRNA expression of COX2, PTGES and TSG6. Expression levels were measured with quantitative real-time PCR and normalized to those of HPRT1 following total RNA isolation 24 h post stimulation from MSC seeded at 5.2*10^3^ cells/cm^2^ and stimulated with 0.2 or 10 ng/ml IL1beta or left untreated. B) TSG6 ELISA with supernatants 24 h post stimulation from MSC seeded at 5.2*10^3^ cells/cm^2^ and stimulated with 0.2 or 10 ng/ml IL1beta or left untreated. C) PGE^2^ detection with the Biotrend PGE2 Enzyme Immunoassay Kit in supernatants 24 h post stimulation from MSC seeded at 5.2*10^3^ cells/cm^2^ and stimulated with 0.2 or 10 ng/ml IL1beta or left untreated. Scatter plots and mean from up to 7 donors are presented. Significance of regulation was determined with one-way ANOVA followed by a Dunnett’s post-test; *p<0.05.
(TIF)

S4 Supporting Information Influence of the 0.2 or 10 ng/ml IL1beta on the expression of 84 inflammatory factors in MSC. Total mRNA was isolated 24 h post stimulation from MSC seeded at 5.2*10^3^ cells/cm^2^ and stimulated with 0.2 ng/ml, 10 ng/ml IL1beta or left untreated. Quantitative real-time PCR with the RT2 Profiler PCR Array Human Inflammatory Response was performed. Analysis was performed with the RT Profiler PCR Array Data Analysis version 3.5 online (http://pcrdataanalysis.sabiosciences.com/pcr/arrayanalysis.php), which uses a Student’s t-test to determine significant gene expression fold changes in stimulated MSC compared to controls, p<0.05. Dots represent the mean from 3 independent donors. Genes that were significantly and more than two-fold upregulated in both PTC and IL1beta treated MSC are marked with their gene name.
(TIF)

S5 Supporting Information Complete array data to Fig. 4 and S2 Supporting Information depicting the influence of the PTC, 0.2 and 10 ng/ml IL1beta on the expression of 84 inflammatory factors in MSC. Total mRNA was isolated 24 h post stimulation from MSC seeded at 5.2*10^3^ cells/cm^2^ and stimulated with the PTC, 0.2 ng/ml, 10 ng/ml IL1beta or left untreated. Quantitative real-time PCR with the RT2 Profiler PCR Array Human Inflammatory Response was performed. Analysis was performed with the RT Profiler PCR Array Data Analysis version 3.5 online (http://pcrdataanalysis.sabiosciences.com/pcr/arrayanalysis.php), which uses a Student’s t-test to determine significant gene expression fold changes in stimulated MSC compared to controls.
(PDF)
