# Supplementary figures and images for: Crucial Role of IL1beta and C3a in the In Vitro-Response of Multipotent Mesenchymal Stromal Cells to Inflammatory Mediators of Polytrauma
Source: PLoS One. 2015 Jan 6;10(1):e0116772. doi: 10.1371/journal.pone.0116772 (PMC4285554; doi:10.1371/journal.pone.0116772)

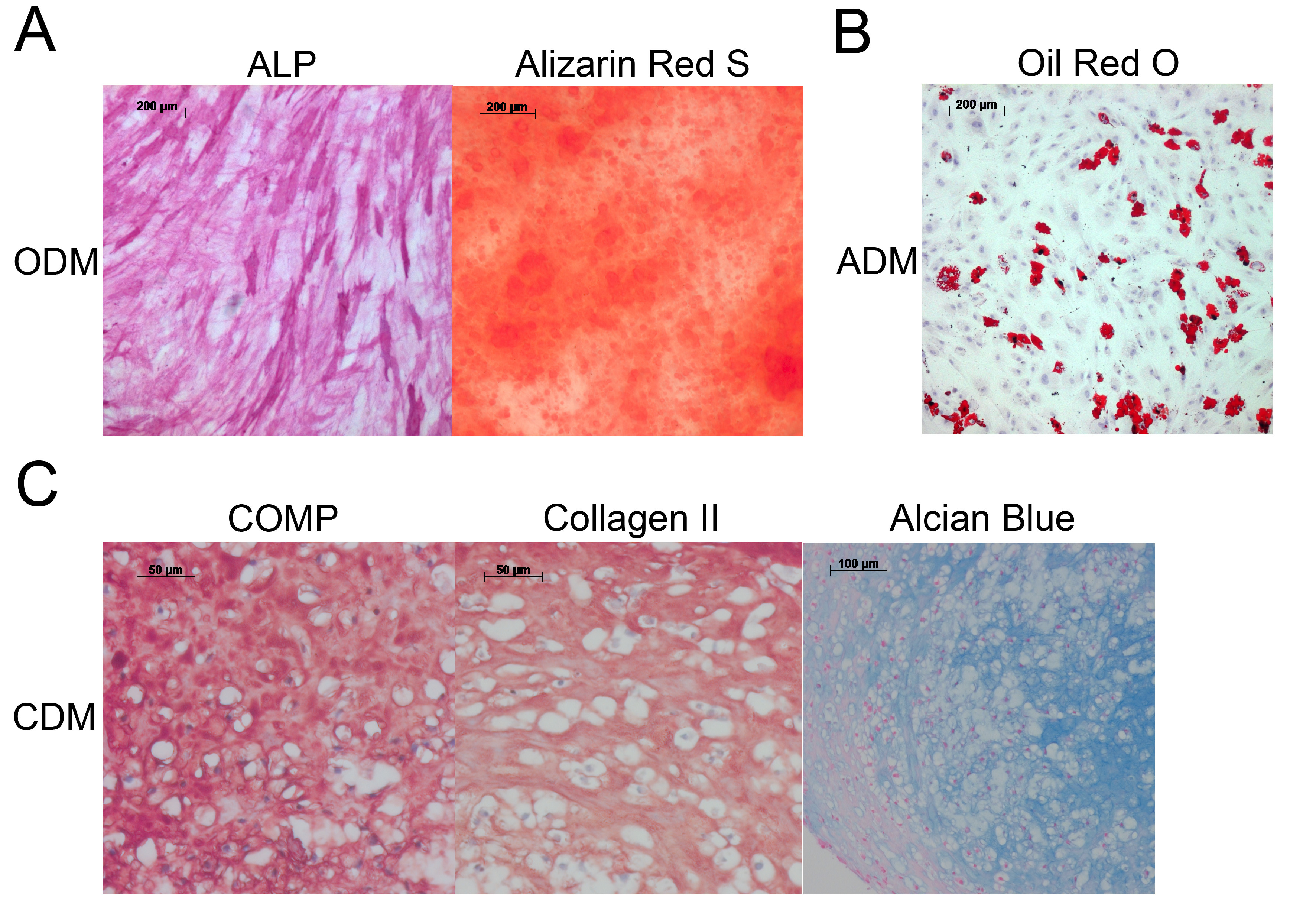

Supplement: S1 Supporting Information — MSC were cultured with A) osteogenic differentiation medium (ODM), B) adipogenic differentation medium (ADM) or C) chondrogenic differentiation medium (CDM). A) Positive osteogenic differentiation as evidenced by positive staining for ALP (red dye deposit) and Alizarin S (red: calcium deposition). B) Positive Oil Red O staining indicates adipogenic differentiation (red: stained lipid droplets). C) Positive chondrogenic differentiation as demonstrated by positive immunohistochemical staining for cartilage oligomeric matrix protein (COMP) and collagen type II and positive Alcian Blue staining. (JPG) [file pone.0116772.s001.jpg]

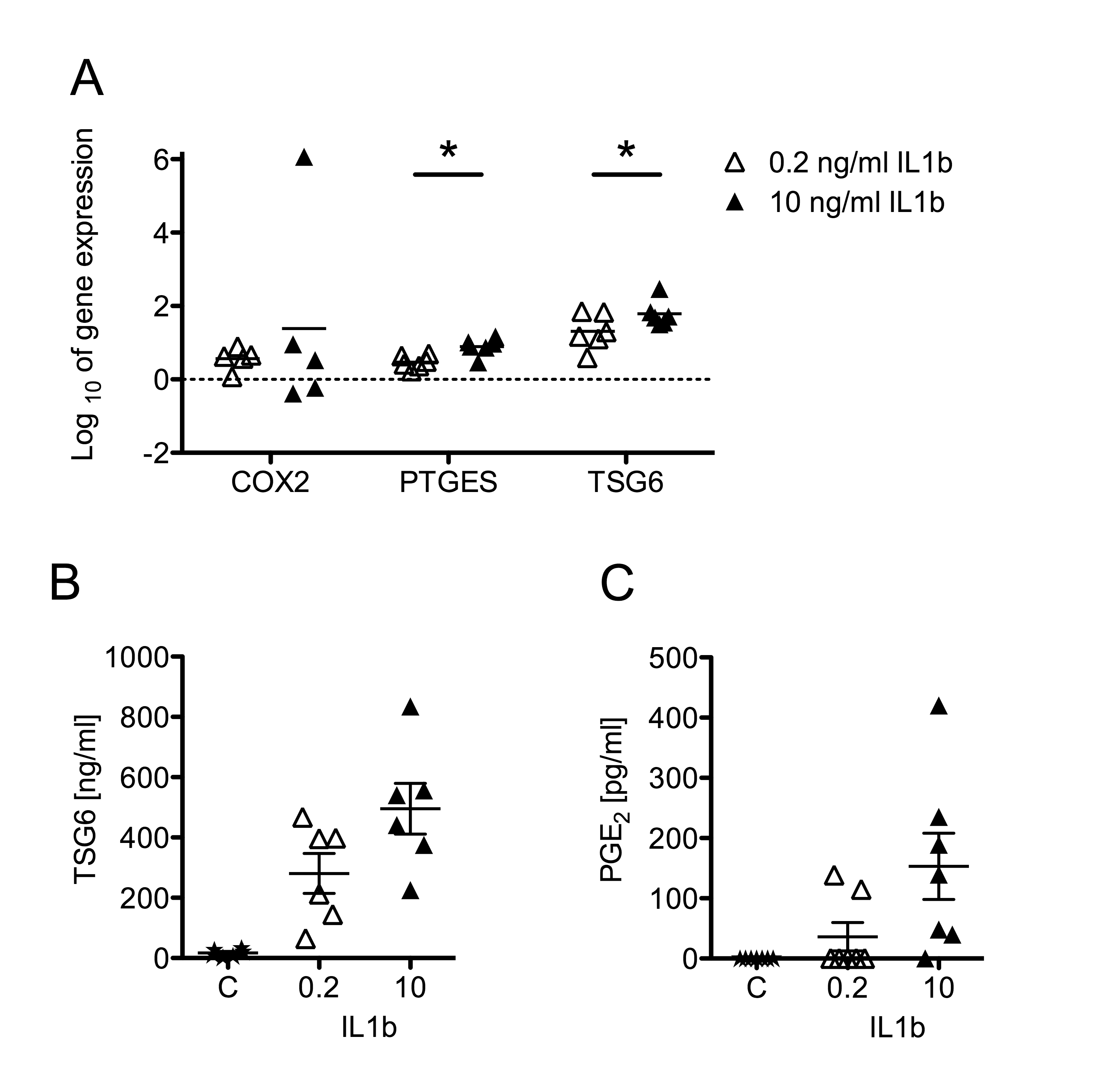

Supplement: S3 Supporting Information — A) mRNA expression of COX2, PTGES and TSG6. Expression levels were measured with quantitative real-time PCR and normalized to those of HPRT1 following total RNA isolation 24 h post stimulation from MSC seeded at 5.2*103 cells/cm2 and stimulated with 0.2 or 10 ng/ml IL1beta or left untreated. B) TSG6 ELISA with supernatants 24 h post stimulation from MSC seeded at 5.2*103 cells/cm2 and stimulated with 0.2 or 10 ng/ml IL1beta or left untreated. C) PGE2 detection with the Biotrend PGE2 Enzyme Immunoassay Kit in supernatants 24 h post stimulation from MSC seeded at 5.2*103 cells/cm2 and stimulated with 0.2 or 10 ng/ml IL1beta or left untreated. Scatter plots and mean from up to 7 donors are presented. Significance of regulation was determined with one-way ANOVA followed by a Dunnett’s post-test; *p<0.05. (TIF) [file pone.0116772.s003.tif]

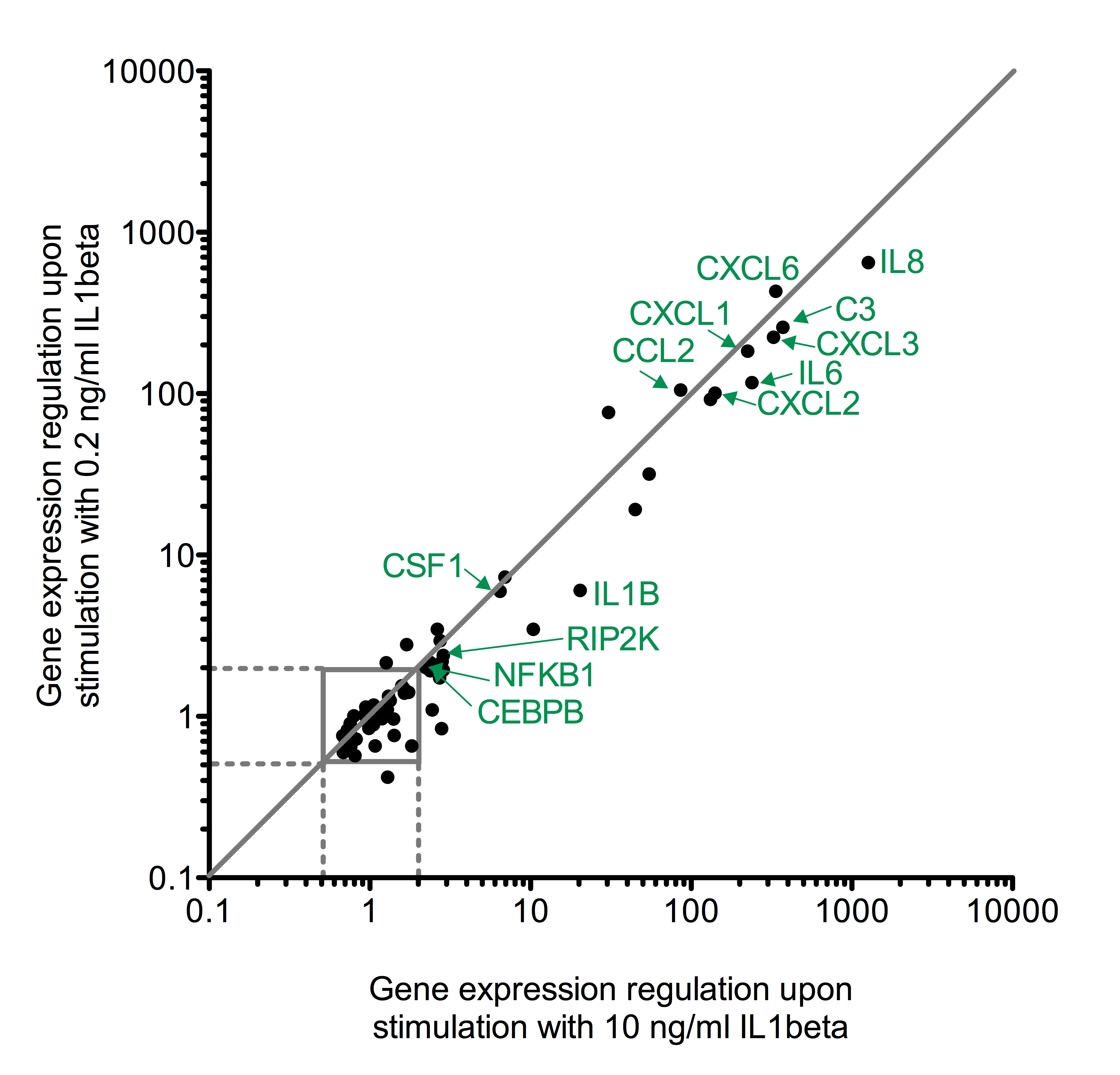

Supplement: S4 Supporting Information — Total mRNA was isolated 24 h post stimulation from MSC seeded at 5.2*103 cells/cm2 and stimulated with 0.2 ng/ml, 10 ng/ml IL1beta or left untreated. Quantitative real-time PCR with the RT2 Profiler PCR Array Human Inflammatory Response was performed. Analysis was performed with the RT Profiler PCR Array Data Analysis version 3.5 online (http://pcrdataanalysis.sabiosciences.com/pcr/arrayanalysis.php), which uses a Student’s t-test to determine significant gene expression fold changes in stimulated MSC compared to controls, p<0.05. Dots represent the mean from 3 independent donors. Genes that were significantly and more than two-fold upregulated in both PTC and IL1beta treated MSC are marked with their gene name. (TIF) [file pone.0116772.s004.tif]
