# Supplementary material for: Crucial Role of IL1beta and C3a in the In Vitro-Response of Multipotent Mesenchymal Stromal Cells to Inflammatory Mediators of Polytrauma
Source: PLoS One. 2015 Jan 6;10(1):e0116772. doi: 10.1371/journal.pone.0116772 (PMC4285554; doi:10.1371/journal.pone.0116772)
Supplement: S2 Supporting Information — Exemplary percentages of marker carrying MSC from 2 donors are depicted. (PDF) [file pone.0116772.s002.pdf]

CD marker	% positive	
	donor 1	donor 2
CD45	0.3	0.9
CD90	99.0	98.3
CD73	99.5	98.5
CD105	99.6	99.5
CD29	99.7	99.9
CD166	99.2	99.8
