# Supplementary material for: Crucial Role of IL1beta and C3a in the In Vitro-Response of Multipotent Mesenchymal Stromal Cells to Inflammatory Mediators of Polytrauma
Source: PLoS One. 2015 Jan 6;10(1):e0116772. doi: 10.1371/journal.pone.0116772 (PMC4285554; doi:10.1371/journal.pone.0116772)
Supplement: S5 Supporting Information — Total mRNA was isolated 24 h post stimulation from MSC seeded at 5.2*103 cells/cm2 and stimulated with the PTC, 0.2 ng/ml, 10 ng/ml IL1beta or left untreated. Quantitative real-time PCR with the RT2 Profiler PCR Array Human Inflammatory Response was performed. Analysis was performed with the RT Profiler PCR Array Data Analysis version 3.5 online (http://pcrdataanalysis.sabiosciences.com/pcr/arrayanalysis.php), which uses a Student’s t-test to determine significant gene expression fold changes in stimulated MSC compared to controls. (PDF) [file pone.0116772.s005.pdf]

Gene Name	PTC Fold Change	p-Value	0.2 ng/ml IL1b Fold Change	p-Value	10 ng/ml IL1b Fold Change	p-Value
IL8	935.5832	0.000001	649.4414	0.000447	1269.2163	0.000289
CXCL6	556.6226	0.002538	430.7048	0.001839	336.3662	0.048848
C3	335.0089	0.001624	258.0289	0.000801	372.5755	0.000004
CXCL3	323.9713	0.000376	223.8501	0.006314	325.4718	0.004529
CXCL1	245.0992	0.000013	183.617	0.001649	224.4975	0.007474
CXCL5	167.8919	0.04321	92.074	0.053091	132.03	0.010926
IL6	166.9249	0.009034	116.9479	0.001017	238.8105	0.014677
CXCL2	125.3417	0.000682	100.8725	0.002429	141.0167	0.007164
CCL2	107.8629	0.003789	105.1561	0.003414	86.2063	0.026496
CXCL10	78.9603	0.105352	76.447	0.112145	30.6197	0.195951
CCL8	44.9595	0.054343	31.7912	0.028959	54.7159	0.093791
CCL5	36.2453	0.013579	19.156	0.041189	44.8299	0.061724
IL1B	18.4501	0.000253	6.0303	0.004973	20.3773	0.049768
CCL7	9.1825	0.021877	7.305	0.039448	6.927	0.064521
CSF1	6.6794	0.0136	5.9507	0.027727	6.4668	0.033704
IL1A	6.1998	0.039571	3.4635	0.162601	10.4027	0.020162
TLR3	3.6336	0.031821	3.4695	0.027354	2.6294	0.177186
TLR2	3.2241	0.084592	2.9463	0.070467	2.7363	0.195736
CXCL9	2.9943	0.230963	2.192	0.37268	2.8197	0.275829
RIPK2	2.6324	0.005529	2.389	0.003729	2.8674	0.004277
C3AR1	2.3304	0.123244	1.9326	0.178117	2.869	0.017881
CD40	2.3304	0.111596	1.3921	0.615088	1.6402	0.321454
IL1R1	2.2824	0.07398	2.1593	0.076913	2.3793	0.071496
CEBPB	2.1568	0.000274	2.0077	0.002686	2.2329	0.046973
NFKB1	2.0054	0.002882	1.9148	0.010099	2.3793	0.000064
C4A	1.9159	0.066011	1.5455	0.322788	1.5816	0.180265
MYD88	1.7059	0.037569	1.4312	0.132424	1.6402	0.100048
CCL13	1.5652	0.543005	2.1531	0.149077	1.2626	0.939451
TNF	1.5643	0.474025	1.7317	0.477467	2.7174	0.146222
CCL11	1.5598	0.542554	2.7857	0.137129	1.6951	0.567374
LY96	1.5304	0.030155	1.4082	0.09422	1.7418	0.007443
CCL3	1.4765	0.221497	1.0985	0.784752	2.4434	0.102054
BCL6	1.4537	0.640718	1.4842	0.588589	1.6279	0.454726
IL1RN	1.4312	0.742833	0.8412	0.627348	2.797	0.151031
FLT3LG	1.4304	0.066025	1.3346	0.189229	1.3071	0.282939
ITGB2	1.2359	0.766163	1.256	0.752509	1.3369	0.981559
TLR4	1.2105	0.419549	1.1216	0.645204	1.0709	0.834192
IL10RB	1.1835	0.1978	1.0447	0.781704	1.1248	0.460461
TLR5	1.1565	0.874143	1.0161	0.867269	0.9307	0.678275
TIRAP	1.1491	0.182877	1.1438	0.220403	0.9442	0.975479
IL23A	1.0878	0.791418	0.9669	0.854108	1.1876	0.495227
TOLLIP	1.0747	0.67404	1.0357	0.885645	0.9356	0.69568
IL6R	1.0465	0.949079	1.1746	0.63417	1.0586	0.713814
TLR1	1.0405	0.771544	0.7239	0.052503	0.8201	0.624785
IL1RAP	1.0381	0.651749	0.9619	0.657041	1.405	0.221753
HDAC4	1.0363	0.827516	0.9232	0.75311	0.958	0.831497
TLR6	0.9992	0.748608	1.0062	0.741739	0.7949	0.533445
CCR1	0.9906	0.691723	0.8215	0.615311	0.7252	0.539721
NR3C1	0.9843	0.86077	0.9442	0.680499	0.9708	0.802987
IL18	0.9259	0.651677	0.7608	0.833864	1.4197	0.395014
TNFSF14	0.834	0.548361	0.9042	0.620082	0.7551	0.487199
CCL19	0.7494	0.602362	0.6172	0.51585	0.7156	0.507798
LTB	0.7349	0.52287	0.8902	0.655543	1.0513	0.849879
IL10	0.7206	0.547117	0.7687	0.546032	0.7617	0.577668
IL9	0.7197	0.513563	0.6547	0.561042	1.822	0.384353
CCL4	0.7197	0.513563	0.6547	0.561042	1.0784	0.739262
CCL16	0.7197	0.513563	0.6547	0.561042	0.759	0.548497
CCL17	0.7197	0.513563	0.6547	0.561042	0.759	0.548497
CCL22	0.7197	0.513563	0.6547	0.561042	0.759	0.548497
CCR2	0.7197	0.513563	0.6547	0.561042	0.759	0.548497
CCR3	0.7197	0.513563	0.6547	0.561042	0.759	0.548497
CCR4	0.7197	0.513563	0.6547	0.561042	0.759	0.548497
CD40LG	0.7197	0.513563	0.6547	0.561042	0.759	0.548497
CRP	0.7197	0.513563	0.6547	0.561042	0.759	0.548497
CXCR4	0.7197	0.513563	0.6547	0.561042	0.759	0.548497
FASLG	0.7197	0.513563	0.6547	0.561042	0.759	0.548497
IFNG	0.7197	0.513563	0.6547	0.561042	0.759	0.548497
IL18RAP	0.7197	0.513563	0.6547	0.561042	0.759	0.548497
IL1F10	0.7197	0.513563	0.6547	0.561042	0.759	0.548497
IL22	0.7197	0.513563	0.6547	0.561042	0.759	0.548497
IL22RA2	0.7197	0.513563	0.6547	0.561042	0.759	0.548497
IL23R	0.7197	0.513563	0.6547	0.561042	0.759	0.548497
CXCR1	0.7197	0.513563	0.6547	0.561042	0.759	0.548497
CXCR2	0.7197	0.513563	0.6547	0.561042	0.759	0.548497
KNG1	0.7197	0.513563	0.6547	0.561042	0.759	0.548497
LTA	0.7197	0.513563	0.7082	0.592167	0.759	0.548497
TLR7	0.7013	0.528771	0.6634	0.538551	0.698	0.593537
CCR7	0.6634	0.447917	1.103	0.758727	1.2847	0.931546
FOS	0.6596	0.383884	0.7595	0.385149	0.6797	0.413135
CCL21	0.6475	0.442428	0.5986	0.519363	0.6829	0.476009
NOS2	0.6287	0.457553	0.5719	0.507535	0.8088	0.897295
CCL24	0.6158	0.450728	0.6509	0.516511	0.7477	0.557992
CCL23	0.4505	0.459714	0.4194	0.54414	1.2891	0.947368
NFATC3	0.2278	0.213311	0.8422	0.136185	0.9855	0.942494
